# Biosafety and Biosecurity in European Containment Level 3 Laboratories: Focus on French Recent Progress and Essential Requirements

**DOI:** 10.3389/fpubh.2017.00121

**Published:** 2017-05-31

**Authors:** Boris Pastorino, Xavier de Lamballerie, Rémi Charrel

**Affiliations:** ^1^UMR “Emergence des Pathologies Virales” (EPV: Aix-Marseille Univ – IRD 190 – Inserm 1207 – EHESP – IHU Méditerranée Infection), Marseille, France; ^2^UMR 190 “Emergence des Pathologies Virales”, Virology, 19-21 bd jean moulin faculté de medecine de la timone, Institut hospitalo-universitaire Méditerranée infection, APHM Public Hospitals of Marseille, Marseille, France

**Keywords:** biosafety, BSL-3, European union, regulations, infectious disease transmission, vertical, containment level 3, France

## Abstract

Even if European Union (EU) Member States are obliged to implement EU Directives 2000/54/EC *on the protection of workers from risks related to exposure to biological agents at work*, national biosafety regulations and practices varied from country to country. In fact, EU legislation on biological agents and genetically modified microorganisms is often not specific enough to ensure harmonization leading to difficulties in implementation for most laboratories. In the same way, biosecurity is a relatively new concept and a few EU Member States are known to have introduced national laboratory biosecurity legislation. In France, recent regulations have reinforced biosafety/biosecurity in containment level 3 (CL-3) laboratories but they concern a specific list of pathogens with no correlation in other European Members States. The objective of this review was to summarize European biosafety/biosecurity measures concerning CL-3 facilities focusing on French specificities. Essential requirements needed to preserve efficient biosafety measures when manipulating risk group 3 biological agents are highlighted. In addition, International, European and French standards related to containment laboratory planning, operation or biosafety equipment are described to clarify optimal biosafety and biosecurity requirements.

## Introduction

Recent international events such as successive outbreaks (West Nile virus in North America, Chikungunya virus in the Indian Ocean and in the New World, Ebola virus in West Africa, and Zika virus in the New World) and the concomitant fear of bioterrorism have stimulated a growing reinforcement of biosecurity and biosafety measures (European Union Chemical, Biological, Radiological, and Nuclear action plan B2, March 2014) (1). As a direct consequence, European laboratories in which infectious microorganisms are manipulated were confronted to a drastic increase in biosafety/biosecurity regulations. Useful information is available in websites of the agencies in charge of specific aspects, however, its diversity led to difficulties to comply with all these rules. Moreover, regulations can be different in European Union (EU) Member State countries and despite efforts of national agencies to place online specific information, European laboratories working with infectious pathogens are confronted to a large number of complex rules and regulations.

This review aims at compiling the principal concepts of European academic containment level 3 (CL-3) laboratory management, biosafety, and biosecurity based on applicable EU Directives and French regulations. It also highlights essential information to ensure workers and environment protection according to commonly recommended biosafety measures.

As mentioned in the World Health Organization (WHO) Laboratory biosafety manual (2), “Laboratory biosafety” is the term used to describe the containment principles, technologies, and practices that are implemented to prevent unintentional exposure to pathogens and toxins or their accidental release. “Laboratory biosecurity” refers to institutional and personal security measures designed to prevent the loss, theft, misuse, diversion, or intentional release of pathogens and toxins.

Surveillance of laboratory-acquired infection (LAI) is, therefore, an efficient marker to evaluate the effectiveness of biosafety and to optimize the risk assessment in CL-3 laboratories (3–5). Before the era of containment laboratories, the 10 microorganisms responsible for >50% of LAI were brucellosis, Q fever, viral hepatitis, typhoid fever, tularemia, tuberculosis, dermatomycoses, Venezuelan equine encephalitis, psittacosis, and coccidioidomycosis (6). Byers and Harding reported that 85% of LAI were caused by *Mycobacterium tuberculosis, Coxiella burnetii*, hantaviruses, arboviruses, hepatitis B and C viruses, *Brucella* spp., *Salmonella* spp., *Shigella* spp., and *Cryptosporidium* spp. (7). In the USA, from 2004 to 2010, only 11 LAIs were reported to CDC for microorganisms listed as Biological Select Agents and Toxins (8): six cases due to *Brucella* spp., four cases due to *Francisella tularensis*, and one case due to *Coccidioides immitis/posadasii* (9). Although there is no harmonized system for the reporting of laboratory incidents and accidents at the EU level, few LAIs have been described in European laboratories during the last decade highlighting a drastic reduction of these accidents in CL-3 laboratories (5). Doubtless, current practices have also minimized worker’s pathogen exposition and improvements in containment equipment, engineering controls, and safety training contributed greatly to this reduction.

It is known that about 80% of LAIs are caused by inhalation (particularly by aerosols) or direct contact between contaminated surfaces (gloves and hands). The other routes of infection are percutaneous inoculation (needlestick injuries, broken glass injury, and/or animal bites or scratches) and LAIs due to smoking eating, or accidental aspiration through a pipette has now disappeared because of banishment of these practices (3, 10). Actually, the risk assessment related to microorganisms manipulated in CL-3 laboratories has to consider the possible route of transmission as well as the minimal infective dose for humans (11). Consequently, specific biosafety measures in addition to general conception of laboratory facilities must be enforced (12).

## Pathogens Classification, Biosafety Level, and Biosecurity

The WHO has recommended to classify microorganisms according to four general risk groups (RG1–RG4) depending on the severity of the natural disease, the route of infection, and the therapeutic and preventive arsenal. RGs reflect the risk for laboratory workers and for the community; they relate but do not equate with the confinement level in which pathogens must be manipulated (2). When a specific RG is attributed to a given microorganism, it must be manipulated in laboratory enforcing the same containment level (CL). CL defines a set of biocontainment measures to isolate dangerous biological agents in an enclosed laboratory facility. There are four CLs: CL-1 to CL-4 (13). Poliovirus (PV) is a typical example of biorisk management: in 2015, a revised edition of the WHO Global Action Plan (GAP) to minimize “PV facility-associated risks after type-specific eradication of wild PVs and sequential cessation of oral PV vaccine use (GAPIII)” was implemented (14, 15). Containment of PVs, as laid out in GAPIII, is taking place in three phases linked to global milestones in polio eradication. Current (Phase 1) activities are focused on containment of type 2 wild PV or vaccine-derived PV, as well as on preparation for containment of vaccine PVs of type 2 (OPV/Sabin2). In 2016, the European Region has completed the first step, requiring Member States to (i) provide national inventories of all facilities hosting wild PVs (WPVs) and (ii) destroy all unneeded WPV2 materials or designate a PV essential facility (PEF) and accordingly a national authority for containment tasked with national certification of the PEF.

In 2000, EU has published RG/CL classification in the Directive No. 2000/54/EC “on the protection of workers from risks related to exposure to biological agents at work.” Moreover, many countries within Europe use additional containment measures based on specific Directives [for example, Directive 2009/41/EC for genetically modified microorganisms (GMMs) or Directive 2003/85/EC relating to Foot and Mouth Disease Virus]. Although National and EU classifications are globally congruent, there are still discrepancies that render the situation quite complex. In addition, there are sometimes some differences between classifications issued from national agencies within the same country (for example, pathogen classification list given by the French High Council of Biotechnologies (HCB) 2013 guideline and those elaborated from the French specific law updated in 1998) or Member States (for example, Omsk hemorrhagic fever and Kyasanur Forest disease viruses classified RG4 in United Kingdom and RG3 in France).

### Bioterrorism Issue

Since 1999, the US Centers for Disease Control and Prevention (CDC) defined three groups of potential bioterrorism biological (A, B, and C) and the European Commission formed a task force on bioterrorism, which became operational in May 2002 and defined two groups of potential bioterrorism biological agents, high and very high threat.^1^ Similarly, Russia evaluated the potential bioterrorism agents and identified three groups of potential bioterrorism agents (groups 1, 2, and 3) (13). From 2001, France has implemented a regulation system to register facilities possessing substances from the list of the microorganisms and toxins (MOTs). The scope of the legislation was the control of MOTs use which was likely to be a public health risk and the new regulatory framework was applicable to all French laboratories involved in any operation using MOTs for diagnostic, research, development, or teaching purposes. The list mentions high-risk agents/toxins that have been determined to have potential for use as bioweapons and were subject to more drastic measures concerning their acquisition and handling (French Decree of June 30, 2010). It is important to specify that this list included genetic material (DNA or RNA) less than 500 nucleotides in length or toxins, protein toxin fragments containing fewer than 167 amino acids. The extension of such regulation concerning non-infectious materials is unique to France. These measures were enforced in French academic laboratories with an update list of pathogens classified as MOT in the order of April 30, 2012 (French Order of April 30, 2012). Every year, laboratories have to report to the Agence national de Securité du Médicament et des Produits de Santé (ANSM, the French Biosafety/Biosecurity Agency) an inventory of MOT samples. Legal MOT obligations led to additional difficulties when animal experiments are planned with these pathogens knowing that corresponding A-3-facility (for “Animal facility-level 3”) and biologists have to follow the same regulations. Moreover, French laboratories using MOT pathogens were also identified as ZRR (for “Zone à Régime Restrictif”; restricted access area) area. This ZRR classification was based in the order of July 3, 2012, which defined four risks for the nation (R1–R4): (i) the risk of violation of economic interests (R1); (ii) the risk of abuses of defense capabilities (R2); (iii) the risk of proliferation (R3); and (iv) the risk of terrorism (R4). Under institutional supervision, the “director of such ZRR laboratories (which may be subject to criminal sanction for not complying with the law and instructions) must set up the monitoring of any person who wishes to join a research team and therefore work inside the research unit” (16).

## CL-3 Laboratory: Design, Construction, and Biosafety/Biosecurity Rules

### Design and Construction of CL-3 Facilities

There is currently no harmonization at the European level for guiding CL-3 laboratories construction. Annex V of the EU Directive 2000/54/EC gives some indications concerning containment measures and CLs but they are not sufficiently precise to ensure harmonization for all European CL-3 laboratories (17). Some countries (France, United Kingdom, Germany, etc.) have adopted regulations, rules, or guidelines, and there are several ISO/EN standards available in the EU that can be applied for containment laboratory planning, construction, and operation. In addition, European standards have been developed for biosafety equipment, e.g., autoclaves, biosafety cabinets (BSCs), and personal protective equipment (PPE), but regular oversight and recertification are guided by national specifications (17).

A CL-3 laboratory has special engineering and design features identified through national regulatory agencies or described in specific international guidelines: at the heart of biosafety is the containment of hazardous agents through multiple levels of barriers. For example, the French order of July 16, 2007 defined some minimal requirements for CL-3 facilities.

Direct protection against pathogens consists of PPE that are worn by the worker and intend to protect himself from direct contact with the infectious agents manipulated in the laboratory. PPE consist of gloves, gowns, masks, respiratory protection, and positive-pressure ventilation suits as well as the use of good laboratory practices.

Primary containments or BSCs are unique, thus hardly classifiable, since they intend to separate the hands and forearms [in direct contact with the worker from the rest of the body (outside of the cabinet)] by generating an air-barrier preventing the microorganism to escape from the inside of the cabinet into the environment.

Secondary containments consist of devices (i) preventing or mitigating the presence of pathogens within the CL-3 environment and (ii) avoiding pathogens to exit the CL-3 containment zone in order to protect the outside of CL-3 environment from what is contained and manipulated within the CL-3.

Secondary containments consist of facility design with air-tight rooms; air handling and filtration [the laboratory building supply/exhaust air should be high-efficiency particulate air (HEPA) filtered]; air locks; showers; laundry; sewerage treatment; waste disposal; sterilizers; redundant services and equipment; and material finishes.

Tertiary barriers deal with the physical operation with items such as walls, fences, security, and animal exclusion zones (2, 12, 18).

Europe has no specific recommendations about facility air change rates other than a German DIN standard that is generally followed by central Europe. This standard is DIN 1946–1947 “Ventilation and air conditioning—Part 7: Ventilation systems in laboratories” and includes a recommendation but not a requirement for minimum lab ventilation of 25 m^3^/h/m^2^. This minimum ventilation flow recommendation corresponds to about 9.1 air change hour (ACH) for a 9-foot ceiling or an 8.2 ACH rate for a 10-foot ceiling space. Based on risk assessment and general practices, air change rates are often set to a single value between 6 and 12 ACH for most CL-3 laboratories. Ventilation rates in animal facilities usually range 10–15 ACH. The laboratory quality stepwise implementation tool (WHO, 2015) recommended a minimum of six ACH for CL-3 facilities. This minimum air flow shall be maintained permanently, whether the laboratory is used for experiments or not. Ventilation rates were established not only for efficient prevention of airborne contamination but also for odor control in the laboratory air space. Airflow in CL-3 facilities shall be designed to move from “clean” areas toward the biocontainment space. A negative pressure differential of 12.5 Pa (0.05 in w.g.) must be maintained between each pressure zone following the laboratory quality stepwise implementation tool (WHO, 2015) or US recommendations (19). For CL-3 laboratories containing multiple zones, greater negative pressure must be established in high-risk rooms. For ensuring the pressure differential in all containment rooms, specific monitoring and control devices shall be provided as well as visual readout, alarm devices at the entrance of the containment space, in anterooms, or at entrance of the individual rooms within the containment suite. In fact, multiple possible designs exist for CL-3 laboratories (12, 20), which facilities should be verified at least annually with the minimum facility verification requirements described in Table 1.

**Table 1:** **Minimum containment level 3 (CL-3) facility verification requirements (http://www.emerge.rki.eu/Emerge/EN/Content/Topics/Rules/ECL_Biorisk.pdf?__blob=publicationFile)**.

Minimum facility verification requirements (annual or regular control)	Critical equipment that should be operational and regularly test in case of power failure	Changes for which retesting may be required
Differential pressure	Primary or secondary containment	Replacement of CL-3 fans, duct, air valves
Air flow	Communication system	Replacement or repair of control wiring
Air flow turnover	Access security systems and limitations	Changes in building control sequences
High-efficiency particulate air filter certified (photometric control filters in the extraction)	Respirators, space suits	Structural changes
Environmental parameters (noise, temperature, humidity, lighting)	Sensors, alarms	Frequent failures of HVAC system/inoperable HVAC alarms
		Supply exhaust interlocking system failure/reversals of airflow under normal conditions
Autoclaves and other pressure vessels		
Standby power and UPS systems		
Operation of interlocking doors		
Operation of decontamination systems (e.g., autoclaves, fumigation chambers, liquid effluent)		
Liquid effluent treatment systems		
Battery driven emergency lights		

One of the common essential features of CL-3 laboratory includes proper procedures for disposal of biomedical waste and even if an autoclave facility is recommended, biohazardous waste and liquid effluents should also be decontaminated using only validated chemical treatments.

One other important point concerns biosecurity related to the biocontainment: “many CL-3 installations make a half-hearted attempt at limiting positive pressurization by putting in redundant exhaust fans, but fail to provide a feedback mechanism in the control system to shut the supply system off in the event of total loss of exhaust. As the lab begins to lose its negative pressurization, control mechanisms should be in place to counteract this such as limiting the supply air or shutting it off altogether” (18, 21).

### Materials and Technical Considerations

European Union legislation on biological agents and for GMMs is often not specific enough to ensure harmonization of the implementation on the national level. Accordingly, several EU Member States, like France, United Kingdom, or Germany, have developed their own national guidance based on the EU Directives. In other cases, the varying interpretation of the EU Directives can be supplemented by adopting the USA Biosafety in Microbiological and Biomedical Laboratories or the Canadian guidelines (17).

Control of airborne particulates is critical to protect employees from contact with hazardous materials. BSC and CL-3 structures are equipped with HEPA filters that trap hazardous microorganisms for individual and collective protection; indeed, the risk of infection is linked with the number of particles inhaled (22).

**Figure 1:**
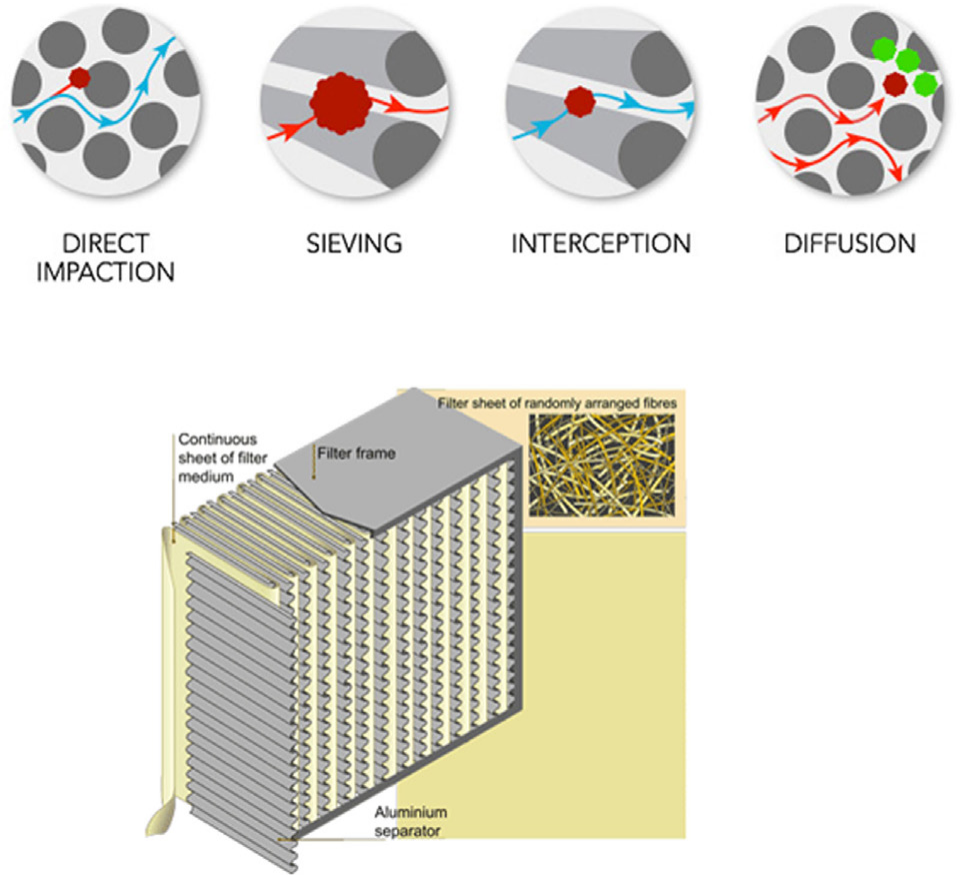
**Description of high-efficiency particulate air filtration**.

High-efficiency particulate air filters are composed of randomly arranged fiberglass with diameters between 0.5 and 2.0 µm and essential factors affecting the filtration are fiber diameter, filter thickness, and face velocity. Even if air space between filter fibers is much greater than 0.3 µm and unlike membrane filters, HEPA filters are designed to target much smaller pollutants and particles. These particles are trapped (they stick to a fiber) through a combination of the following four mechanisms (interception, impaction, diffusion, and sieving) (Figure 1).

The EU standard for both HEPA and ULPA filters classifies filters into different classes depending on their efficiency (EN 1822:2009—test methods for EPA/HEPA/ULPA filters). All EN 1822 specifications are based on a filter’s ability to trap and contain the most penetrating particle size (MPPS) particular to the filter. The MPPS is typically determined by a laser spectrometer or electrostatic classifier and corresponds to the particle size which has most frequently penetrated through the filter. However, aerosolized product of known size is used to control HEPA filters and to determine its efficiency at that specific size.

In Europe, the standard EN 12469 specifies basic requirements for microbiological safety cabinets with respect to safety and hygiene. In French CL-3 laboratories, BSC should be equipped with H14 HEPA filters (99.995% efficient at its MPPS) and selected primarily in accordance with the type of protection needed. BSC should be certified during its installation and regularly according to national or international performance standards and manufacturer’s instructions. To evaluate the cabinet containment, tests should include HEPA filter leaks, down flow velocity profile, face velocity, negative pressure/ventilation rate, air flow smoke pattern, and alarms and interlocks. For CL-3 facilities, HVAC HEPA filtration should be certified annually (2).

### French Biosafety/Biosecurity Rules

The order of July 16, 2007 describes the minimal biosafety measures required for working with RG3 infectious pathogens in French academic laboratories. It sets out the minimal technical preventive measures to be implemented in laboratories conducting research, teaching, analysis, pathological anatomy, and cytology, in autopsy rooms, and in industrial and agricultural facilities. The French pathogen classification and a risk assessment define the required CL for working with infectious agents. However, for pathogens without natural aerosol infection and after a risk assessment, the article 4 states specific situations where RG3 agents can be down classified (working with an attenuated or a vaccine strain, low agent concentration, non-dangerous parasite stage, etc.). For biological samples potentially contaminated by a RG4 virus and in the case of urgent biomedical analysis, the article 5 allows us to perform molecular diagnosis on human samples in a CL-3 academic laboratory. Only, molecular diagnostic can be performed in CL-3, excluding virus isolation and propagation that can only be done in a CL-4 laboratory. Article 5 was enforced during the recent West African Ebola virus outbreak (order of August 6, 2014).

## PPE in CL-3 Laboratories

Primary barriers consist of PPE that are worn by the worker and intend to protect himself from direct contact with the infectious agents manipulated in the laboratory. PPE consist of gloves, gowns, masks, respiratory protection, and positive-pressure ventilation suits as well as the use of good laboratory practices.

European Union Directive 89/656/EEC specifies the minimum health and safety requirements and covers the rules for conformity assessment and placing of PPE on the EU market.^2^ Apart from these, there is no specific EU legislation for CL-3 workers and adapted PPE must be identified based on risk assessment and technical practices.

### Gloves

In CL-3 facilities, gloves act as barriers, protecting persons by reducing the risk of exposure to infectious materials. Moreover, because they were removed and replaced after each manipulation, they also prevent pathogen dissemination in case of hands contamination. The glove selected must conform to EN 374 European standard, and the “Microorganism” pictogram should be present certifying that the glove was conformed to at least a performance level 2 for the penetration test (BS EN ISO 374-1:2016, “Protective gloves against dangerous chemicals and microorganisms. Terminology and performance requirements for chemical risks”). They must also be CE marked for specific use with biological agents. A double gloving strategy is generally the rule since it allows for removal and replacement of the outer glove without exposing the bare skin. Moreover, gloves should be the last piece of PPE to be donned; they must pull over the wrists of the gown. Nitrile gloves are preferred to latex gloves because they can provide better microbiological protection, as well as better protection against chemicals. However, under certain circumstances, powder-free latex gloves could be the better choice (when high degree of sensitivity and dexterity are required) (23).

### Protective Clothing against Biological Hazards

Protective clothing was required in CL-3 laboratory to protect the wearer, against potential contact with infectious substances and avoid germs dissemination. The European standard CSN EN 14126 defines performance requirements for clothing materials to protect against infective agents^3^ (Table 2). The test methods specified in this standard focus on the medium containing the microorganism; such as liquid, aerosol, or solid dust particle. Due to the heterogeneity of microorganisms, the standard does not define performance criteria for specific types of microorganisms. This subtle point needs to be considered in the risk assessment and with reference to the risk group of the infective agent itself but at least the suffix B, the pictogram “protection against biological hazards” and the CE certified as category III should be added. However, independently the EN14126 certification, minimal clothing performance recommended for CL-3 laboratories should be marked 4B (protection against liquid aerosols EN 14605), 5B (protection against airborne solid particulate chemicals pr EN ISO 13982-1), and 6B (limited protection against liquid mist pr EN 13034). A protection against electric arcs (EN 1149-5) was also required and viruses if needed (EN ISO 16604 procedure D). According to EU Directives 89/656/EEC and 89/686/EEC, employers shall be responsible for selecting a suitable protective equipment available for exposed workers. In CL-3 facilities, suits with over taped seams are recommended, since viruses, bacteria, and spores are small enough to penetrate through the openings of sewn seams. These protective suits could not be decontaminated and were specified for “single use only” by manufacturers. However, in practice and after specific training, CL-3 procedures might specify that workers were responsible to their own individual protection and have to define the periodic control allowing the conditions for a reuse of each personal protection.

**Table 2:** **European standard EN 14126 for clothing materials to protect against infective agents**.

Type	Description	Relevant standard
1aB, 1bB, 1cB, 2B	Gas-tight, non-gas-tight	EN 943-1, EN-943-2
3B	Protection against pressurized liquid chemicals	EN 14605
4B	Protection against liquid aerosols	EN 14605
5B	Protection against airborne solid particulate chemicals	Pr EN ISO 13982-1
6B	Limited protection against liquid mist	Pr EN 13034

### Respiratory Protection against Bioaerosols

Based on risk assessment, the use of respiratory protective equipment (RPE) or Class III microbiological safety cabinet was highly recommended in CL-3 laboratory particularly when manipulating specific pathogens (drug-resistant tuberculosis, MERS-CoV, Avian influenza, *Yersinia pestis, Francisella tularensis*, Rift Valley Fever virus, etc.) or for specific experiments (flow cytometry, sonication, freeze drying, infected animals, etc.) (2, 18). Bioaerosol particle size, the airborne agent concentration, and the type of biological agent are the main decision criteria when choosing RPE and every user should be fit tested and trained in the correct use of the respirator. Depending on risk assessment, powered air-purifying respirators (PAPRs) and valved/unvalved disposable respirators were the two types of RPE that could be used in CL-3 confinement.

Powered air-purifying respirators, sometimes called positive-pressure masks, ensured eyes protection and the higher level of protection for airborne pathogens in CL-3 laboratories. The European standard EN12942 (EN 12942:1998 Respiratory protective devices—power-assisted filtering devices incorporating full face masks, half masks, or quarter masks—requirements, testing, and marking) defined PAPRs with full face masks, half masks, or quarter masks and filter classified following the EN 147 standard (TMP1, TMP2, or TMP3) reducing the wearer’s exposure to airborne particles by a factor of 20, 200, or 2,000 (Table 3). Without accident/contamination, PAPRs could be stored and as for protective clothing, CL-3 procedures have to define the periodic control allowing the conditions for their reuse.

**Table 3:** **European standards for powered air-purifying respirators**.

Nominal protection factor				
European device classification	EN 146^a^	EN146/EN12941^b^	EN147^a^	EN147/EN12942^c^
	TH		TMP	
1	10	10	20	20
2	20	50	100	200
3	500	500	2,000	2,000

*^a^Filter classification*.

*^b^Helmet or hood and filter classified following the EN 146 standard (TH1, TH2, or TH3)*.

*^c^Full face masks, half masks, or quarter masks and filter classified following the EN 147 standard (TMP1, TMP2, or TMP3)*.

Both valved and unvalved disposable respirators were tested under the standard EN149 (2001+A1:2009) providing “the minimum requirements for filtering face pieces for protection against particles.” These masks were generally designed for single shift/maximum 8-h use only (marked “NR”) and only two classes could be used in CL facilities:

FFP2: medium filter performance (94% efficiency) reduces the wearer’s exposure to airborne particles by a factor of 10.
FFP3: high filter performance (99.97% efficiency) reduces the wearer’s exposure to airborne particles by a factor of 20.

An FFP2 disposable particulate/filtering half-face piece constituted the minimal protection against RG3 aerosolized pathogens (if particle size is >0.3 µm in diameter) and based on each risk assessment an FFP3 respirator should be usually more adapted and prudent for specific experiments or circumstances.

## Biological Waste Management

### Autoclave Instruction, Training, Maintenance, and Inspection

Biohazardous waste requires inactivation, and steam autoclaving is the universal method for all decontamination processes (2). Country specificities exist; for example, in France, the order of July 16, 2007, requires the presence of an autoclave near or in the technical rooms although when manipulating specific MOTs (Annex 2), the autoclave should have two doors and has to be located exclusively in the technical area (orders of January 23 and June 11, 2013).

As documented in 2010 by the Institute for Reference Materials and Measurements (IRMM),^4^ “The autoclave shall be CE marked in conformity with applicable European Directives and shall be in compliance with the strictest safety and quality standards as foreseen by European regulations”:

– Directive 97/23/EC on pressure equipment.
– Directive 2004/108/EC on electromagnetic compatibility
– Directive 2006/42/EC on machinery safety
– Directive 2006/95/EC on low-voltage devices
– EN 285 on sterilization and large steam sterilizers (where applicable)
– EN 292-294, EN349, EN 418, and/or ISO12100 series on safety of machinery
– EN13445 series on unfired pressure vessels
– EN60204 and EN61010 series on safety requirements for electrical equipment
– EN60601 or 61000 series on electrical equipment and electromagnetic compatibility.

Requirements for qualification of autoclaves can differ according to the country regulation. For example, in France, autoclaves (i) should be calibrated upon installation and declared at the following address: https://authentification.din.developpement-durable.gouv.fr/authSAML/login/ConnectAppli.do, and (ii) specific training should be delivered to the users (French order of March 15, 2000). Qualification should be revalidated every 18 months (order March 15, 2000). Finally, a daily checklist must be validated (Bowie-Dick and Vacuum Leak tests) before use.

In 2010, the European IRMM issued a guide to promote common technical specifications for the supply, installation, and maintenance of steam sterilization autoclaves in CL-3 and CL-2 laboratories.^5^

Basically, in the CL-3 laboratory suite, the autoclave should be a steam sterilization one with external steam supply and any item, device, or solution was considered to be sterile when it was completely free of all living microorganisms and viruses. From an operational standpoint, a sterilization procedure cannot be categorically defined and the optimal cycle parameters for steam sterilization, depended on at least four factors such as size/type load, time and throughput constraints, or steam source. Rather, the procedure was defined to reach a potential surviving of microorganism less than one in one million (10^6^) at the end of the process. However, due to the absence of pertinent standards, biological indicators (BIs) were considered the “gold standard” of load sterilization monitoring. BIs were widely recommended as the preferred device for monitoring and releasing loads. *Geobacillus stearothermophilus* (considered as the most resistant microorganism) was commonly used to validated the sterilization process (spore population reduction up to 4/6 log10 depending upon the laboratory biorisk internal analysis). ISO 14161:2009 provides guidance for BIs efficient utilization in sterilization procedures. When using a CL-3 autoclave it is imperative that pathogens do not escape through the autoclave doors—either *via* poorly designed gaskets or by inadvertently opening the doors simultaneously. Materials and bags should be packed specifically in the chamber to allow easy steam penetration and air removal. Moreover, the autoclave shall be configured to disinfect complex and various waste loads (solids, porous goods, and liquids). CL-3 autoclaves should be carefully specified and equipped with the proper door gaskets and door safety mechanisms. Moreover, all CL-3 autoclaves should be equipped with a biological sealing flange that provided separation and a positive seal between the hazardous side (contained side) and safe side (non-contained side), which is imperative for the safety of personnel. As CL-3 facilities work with microbes that may pose serious health risks, the air/steam effluent had also to be contained, filtered, or decontaminated prior to exhausting into the environment.

## Disinfection and CL-3 Laboratory Decontamination

In recent years, scientific evidence has accumulated about the impact of contaminated surfaces in LAIs (24, 25). The disinfection process and its efficiency depended on the product used but also to its application and the targeted pathogen. The product concentration, its formulation, the water solubility, and pH are important factors as well as the type of surface, soil, the temperature and contact time, humidity, and the mode of product application (with or without mechanical action) (25). Validated methods for surface disinfection and laboratory decontamination are based on risk assessment and finally testing the efficacy of such products in a particular environment and conditions (26). Consequently, no commonly used operating procedures are available for CL-3 facilities. This chapter compiles and reminds essential features to consider in relation with corresponding European or French standards.

### Surface Disinfectants

After each experiment, work surfaces and materials in CL-3 facilities have to be decontaminated. The usual procedure is to spray the disinfectant and wait for a defined time duration depending upon the microorganism manipulated and according to the instructions of the manufacturer. The efficacy of surface disinfectant is based on the mechanisms of action of the active substance with the target organism. The effectiveness of disinfectants depends upon the organism populations present on the surfaces, the concentration of both organisms and disinfectant, the presence of interfering substances (organic material), and the duration of contact. Many disinfectants contain multiple active substances that inactivated microorganisms from reversible processes (disruption of transmembrane proton motive force) or irreversible changes (lysis of the cell for example). In practice, the choice of disinfectant must rely upon each CL-3 lab’s risk assessment and requires to target the microorganisms that are manipulated.

Surface disinfection was defined according to European standard EN 14885. Each manufacturer, independently additional specificities, has to ensure that their authorized disinfectant products meet the requirements described in some or all of the following standards. Examples include the following French or European standards:

- NF T 72-230, NF T 72-231, and NF EN 14347 for sporicidal activity (≥4 log10 of reduction for *Bacillus cereus*, subtilis var. niger, and *Clostridium sporogenes*)
- NF EN 14348 for mycobactericidal activity (≥4 log10 of reduction for *M. avium* and *M. terrae*)
- NF EN 14476 and NF EN 14675 for virucidal activity (≥4 log10 of reduction for adenovirus type 5, PV type 1, norovirus, and *Bovine parvovirus*)
- EN 1650 and EN 1657 + 13624 for fungicidal activity (≥ 4 log10 of reduction for *Aspergillus niger/brasiliensis, Candida albicans*)
- NF EN 1276 and EN 1656+EN 13727 (A1) for bactericidal activity (≥5 log10 of reduction for *Pseudomonas aeruginosa, Staphylococcus aureus, Enterococcus hirae*, and *Escherichia coli*)

### Airborne Surface Disinfection (ASD)

Airborne surface disinfection is used in specific situations such as (i) for decontamination before a periodic maintenance of the laboratory, (ii) before entering or exiting large equipment necessitating handling, (iii) before *in situ* maintenance of a contaminated device or system, or (iv) after accidental spillover of infectious material.

Airborne surface disinfection aims at obtaining microbicidal efficacy against the organisms used in the laboratory. For assessment, surrogates also known as BIs can be selected.

In 2011, the French ASD method (standard NF T 72-281) was proposed to become the European standard, and discussions at the European Committee for Standardization (CEN/TC 216: “Methods of airborne disinfection of surfaces—determination of bactericidal, fungicidal, yeasticidal, sporicidal, and virucidal activity”) are ongoing. For the ASD processes, the latest French standard NF T 72-281 (September 2014) describes a method for determining bactericidal, fungicidal, yeasticidal, sporicidal, and virucidal activity. It applies to automatic and manual processes, non-pressurized (spray type) or pressurized (limited to 10 bar) and a complete ASD methodological guide was published by the French ANSES agency (27).

In terms of performance, the French NF T 72-281 standard describes the minimum log10 reductions expected by the device/product combination. This reduction should be greater than or equal to, respectively:

– 5log for bactericidal activity;
– 4log for fungicidal and virucidal activity;
– 3log for sporicidal activity.

Moreover, these levels of requirements should be adapted to the risks associated with the biological agents handled in each laboratory. Specific revalidation is needed in the case of spatial modification with potential impact on the diffusion or activity of the biocide.

Although formaldehyde is still authorized, the use of other biocides possessing lower toxicity was encouraged (Table 4). As mentioned in the ASD French methodological guide (27), “the level of efficacy of the biocidal products will depend on the diffusion process that is selected and a validation process was inseparable of a “device/product” combination. At least three types of dispersion device are currently available on the market and they are based on the following processes:

- Nebulization: droplet size is less than 5 µm;
- Spraying: droplet size ranges from 10 to 50 µm;
- Flash evaporation: the heated biocidal product (e.g., hydrogen peroxide) vaporizes and is drawn by an airstream into the room.”

**Table 4:** **Example of biocidal products used for airborne surface disinfection (ASD)**.

Product	Forms	Conditions of use	Advantages	Disadvantages	Occupational exposure limits (ppm)^a^
Formaldehyde	Liquid	3–10%	Broad spectrum of activity	Highly irritating, toxic, mutagenic, carcinogenic by inhalation	2
Formaldehyde	Gas	4–10 g/m^3^, 18–22°C and 70% humidity	Broad spectrum of activity	Highly irritating, toxic, mutagenic, carcinogenic by inhalation	2
Glutaraldehyde	Liquid	2%, optimal pH: 8	Broad spectrum of activity	Irritant, toxic to the skin and respiratory tract. Activity greatly reduced in the presence of soiling	0.05
Chlorine derivatives	Liquid	Optimal pH: 6–7	Broad spectrum of activity	Aggressive. Toxic disinfection by-products. Activity reduced in the presence of soiling	0.5
Chlorine dioxide	Gas	Soluble in water	Broad spectrum of activity Unlike hydrogen peroxide gas, it can tolerate a wide range of temperature and humidity	Produced *in situ*, corrosive	0.1
Peracetic acid	Liquid	Relatively unstable: decreases by 0.4% per month	Active at low concentrations in the presence of organic and inorganic soiling	Irritating to eyes and respiratory tract	–
Hydrogen peroxide	Gas–liquid	Useable from 5 to about 35%. Relatively unstable	In fumigation: faster and safer than formaldehyde. More stable than peracetic acid. Greater activity in the gas/liquid form	Depending on the procedure may require humidity to be controlled at low level. Some devices are expensive	1

*ASD methodological guide published by the French ANSES agency (27)*.

*^a^Based on the Directive 2009/161/EU and the British List of approved workplace exposure limits EH/40 (2011). Values for long-term exposure limit: time-weighted average over 8 h*.

In practice, the CL-3 room to be disinfected should be made safe, sealed, and prepared with a pre-cleaning step. The air handling unit should be configuring (stopped or bypass configuration) before the decontamination process and its various phases (biocide dispersion and contact, room aeration).

## RG3 Pathogens Transport

For all types of transport, the recommendations of the Committee of Experts on the Transport of Dangerous Goods (UNCETDG) (a committee of the United Nations Economic and Social Council) are the guidelines for international regulations concerning the transport of infectious substances. These substances are classified and assigned to UN 2814, UN 2900, UN 3291, or UN 3373, as appropriate.

According to the WHO Guidance (28), infectious RG3 pathogens (including infected biological products/animals or medical/clinical wastes) belonged to category A substance with the following characteristics and regulations for transport.

Category A substances were defined as biological material for which exposition can lead to permanent disability, life-threatening, or fatal disease in otherwise healthy humans/animals (UN 2814) or only animals (UN 2900).

### Triple Packaging System

**Figure 2:**
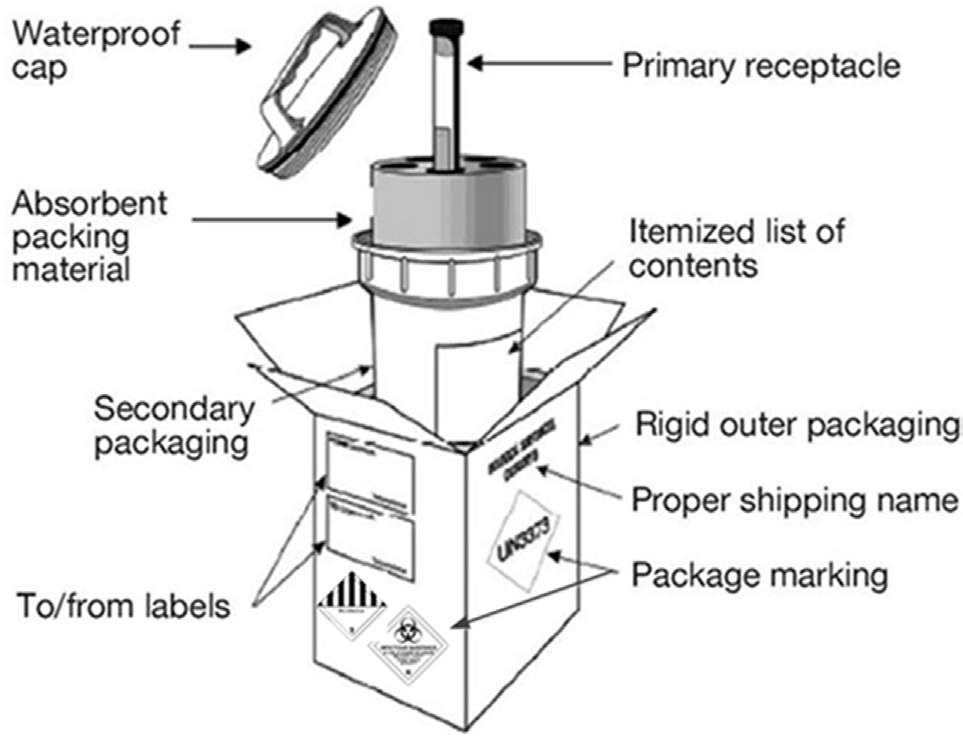
**Basic triple packaging system for infectious substance transport**.

Category A infectious substances have to be transported following the United Nations class 6.2 specifications and Packing Instruction P620. This system must be used for all infectious substances and consisted of three layers (Figure 2). The WHO reference document (28) mentioned “Packages are marked to provide information about the contents of the package, the nature of the hazard, and the packaging standards applied. All markings on packages or overpacks shall be placed in such a way that they are clearly visible and not covered by any other label or marking. Each package shall display the following information on the outer packaging or the overpack:

- the shipper’s (sender’s, consignor’s) name and address
- the telephone number of a responsible person, knowledgeable about the shipment
- the receiver’s (consignee’s) name and address
- the United Nations number followed by the proper shipping name (UN 2814 “INFECTIOUS SUBSTANCE, AFFECTING HUMANS” or UN 2900 “INFECTIOUS SUBSTANCE, AFFECTING ANIMALS only”, as appropriate).
- temperature storage requirements (optional)
- when dry ice or liquid nitrogen is used: the technical name of the refrigerant, the appropriate United Nations number, and the net quantity.

Two types of labels were needed: hazard labels and specific handling ones (infectious substance, non-infectious miscellaneous dangerous substances, dry ice, liquid nitrogen, cryogenic liquid, or orientation labels).

The following shipping documents were required and signed by the shipper:

- for air: the shipper’s Declaration for Dangerous Goods
- an invoice including the receiver’s address plus the number of packages and its contents as well as the weight and value
- an import and/or export permit and/or declaration if needed
- an air waybill for air transport or equivalent documents for road, rail, and sea shipments.

For UN 2814 and UN 2900, a list of contents must be present and placed between the secondary and the outer packaging. Moreover, as specified in the WHO guideline, “when the infectious substances to be transported are unknown, but suspected of meeting the criteria for inclusion in category A and assignment to UN 2814 or UN 2900, the words ‘suspected Category A infectious substance’ shall be shown, in parentheses, following the proper shipping name on the transport document, but not on the outer packaging.”

In addition to regulations previously described, the French National Agency for Medicines and Health Products Safety (ANSM) had to be contacted and delivered authorizations for all operations involving transport, import and export of MOTs.

## Laboratory Biorisk Management and Operating Budgets

The management of biosafety and biosecurity is based on the application of legal obligations and Standard Operational Procedures applicable in many countries. In practice, directors and supervisors of each laboratories are responsible for their implementations to ensure workers, population, or environmental protection (29).

In 2008, the European committee for standardization published a laboratory biorisk management standard CWA 15793 based around the current WHO Biosafety and Biosecurity Guidelines (30). In 2011, an update specifies the requirements for establishing a robust biorisk management system. It provides a comprehensive risk-based approach that takes into account the legal requirements and current knowledge on biosafety and biosecurity. In 2015, a management tool was developed and agreed between 29 European CL-3 laboratories and 6 European CL-4 laboratories working together in the EU-funded Joint Action “Quality Assurance Exercises and Networking on the Detection of Highly Infectious Pathogens.”^6^ Actually, the conversion of CWA 15793 Laboratory Biorisk Management Standard (2011) to the new ISO 35001 (Biorisk Management for Laboratories and Other Related Organizations) is under progress.

The facility operating budget for a high containment facility is the total cost associated with day-to-day operations and maintenance (31). This cost is high due to many considerations that are driven by the need for biosafety/biosecurity. For example, the cost for maintaining a high containment laboratory usually runs in excess of 300% of a non-containment facility even if great variations are possible depending on procedures, type of experiments, or decontamination processes (32). For example, some of the following elements should be budget:

– HEPA filtration (testing and replacement of filters)
– BSCs (annual testing and recertification)
– Specialized waste treatment in addition to autoclaving
– High energy cost for HVAC due to high air change rates
– Annual inspection and testing to confirm the integrity of the facility relative to internal biosafety operating standards
– High-tech systems and equipment for security requirement.

## Regulations to be Applied in France When Working in CL-3 Facilities and MOTs

In France, a law classified the microorganisms for the first time in 1994. In 1997 and 1998, the law was modified to increase the classification of certain pathogens that could constitute a bioterrorism threat. Since 2008, the use of genetically modified organisms (GMOs) in contained spaces for research and educational purposes is subject to prior authorization from the French ministry in charge of research. The ministry must receive the opinion of the French HCB before giving its authorization. Thus, a GMO classification was created in the framework of Directive 2009/41/EC. This list of pathogens was based on previously classification with some updates and modifications in relation to the risks that they present and applying appropriate physical, biological, and chemical containment measures. In fact, some pathogens were classified differently when genetically modified and this lack of harmonization remains unclear (16).

In France, CL-3 laboratories in which MOT are handled must operate ≥12 air changes per hour (ACH). ANSM (the French equivalent of the American Food and Drug Administration) is the National agency to audit and validate CL-3 laboratories. Moreover, since the French Decree of June 30, 2010, specific measures for RG3 agents classified as MOTs were established under the control of ANSM (laboratories conception, biosafety, and biosecurity practices). Although these regulations have improved biologists, population, and environmental protection, they also led to difficulties for many CL-3 laboratories in relation with a large increase in the administrative burden. Moreover, implementation of these new regulations was costly, time-consuming, and generates disparities between CL-3 laboratories working or not with MOTs, since the latter are not subject to systematic control or inspections. All operations involving MOTs, including production, manufacture, transport, import, export, retention, supply, sale, purchase, and use are regulated (33). It was also important to note that French laboratories working on MOTs had to validate their surface or air-borne surface disinfection processes to follow ANSM requirements. Moreover, a CL-3 laboratory using RG3 genetically modified organism needs to obtain a license for their manipulations. This license was granted after an evaluation by the French HCB and must be registered by the police for a validity period of 5 years.

Moreover, in France, specific authorizations and graduates should be obtained for experiments on animals, and CL-3 facilities have to enact several documents:

– Agreement of the structure to practice animal experimentation (renewable each 5 years).
– Agreement of each personal to practice experimentation on animals [two important levels: Level 1 (renewable each 5 years): for experiment responsible and Level 2: for experiment technicians].

All experiments must be submitted beforehand to an ethical committee. The ethical committee, during the evaluation of the experimentation project, gives a particular attention with respect to 3Rs rule (Replacement, Reduction, and Refinement): replace animals each time it is possible, reduce the number of animal used, refinement according to the animal housing, animal transport, animal sacrifice, and reduction of pain (EU 86/609-STE123).

## Conclusion

Regulations in the domain of biosafety and biosecurity have dramatically increased during the last two decades and a clear decrease in LAIs had demonstrated an improvement in the situation. However, progressively, these rules have become more and more complex for people designing or managing CL-3. Moreover, a lack of harmonization of practical guidance ant the development of specific national regulations based on EU Directives (27) led to difficulties in their implementations. French CL-3 laboratories were good examples as they were recently impacted and confronted to exhaustive regulations concerning MOTs without international harmonization. The complexity of the situation to integrate and comply with all these new regulations is not only time- and cost-consuming for laboratories but it can also be counterproductive face to international concurrence. At the same time, there was a growing need to manipulate pathogenic MOTs in academic laboratories for fundamental research purposes, diagnostics, drugs, and vaccines development. However, due to a non-harmonization concerning CL-3 facilities management (design, construction, conformity, and maintenance), it was sometimes difficult for European professional to have a clear and concise overview of essential aspects to consider. This limitation could be problematic not only for new CL-3 laboratories conception (and future substantial cost in case of non-conformity) but also in terms of biosafety and biosecurity if the referential was unclear. Since many years, CL-3 facilities were yet described on international literature but generally these works focused on partial or general considerations with no detailed and practical information. Based on French specificities but also on essential technical aspects and EU Directives, this review resumes biosafety/biosecurity measures concerning CL-3 facilities. Essential CL-3 requirements and French/European standards have been compiled to manipulated safely risk group 3 biological agents in compliance with the legislation. Clarification of optimal biosafety and biosecurity requirements should be useful to any laboratories working with RG3 biological agents in France but also in Europe.

## Author Contributions

All the authors (BP, XL, and RM) contributed in writing the article and approved the final version.

## Conflict of Interest Statement

The authors declare that the research was conducted in the absence of any commercial or financial relationships that could be construed as a potential conflict of interest.

## Footnotes

^1^ec.europa.eu/health/ph_threats/Bioterrorisme/keydo_bio_01_en.pdf.

^2^http://eur-lex.europa.eu/legal-content/EN/TXT/?uri=CELEX:31989L0656.

^3^https://www.en-standard.eu/csn-en-14126-protective-clothing-performance-requirements-and-tests-methods-for-protective-clothing-against-infective-agents.

^4^https://ec.europa.eu/jrc/en.

^5^https://web.jrc.ec.europa.eu/callsfortender/index.cfm?action=app.showdoc&id=7091.

^6^http://www.emerge.rki.eu/Emerge/EN/Content/Topics/Rules/ECL_Biorisk.pdf?__blob=publicationFile.
